# Public contributors' preferences for the organization of remote public involvement meetings in health and social care: A discrete choice experiment study

**DOI:** 10.1111/hex.13641

**Published:** 2022-11-06

**Authors:** Luis E. Loria‐Rebolledo, Verity Watson, Shaima Hassan, Mark Gabbay, Naheed Tahir, Muhammad Hossain, Mark Goodall, Lucy Frith

**Affiliations:** ^1^ Health Economics Research Unit University of Aberdeen Aberdeen UK; ^2^ Department of Primary Care & Mental Health University of Liverpool Liverpool UK; ^3^ National Institute for Health and Care Research ARC North West Coast Liverpool UK; ^4^ Health and Social Care University of Wales Trinity Saint David Carmarthen UK; ^5^ Department of Law, Centre for Social Ethics & Policy University of Manchester Manchester UK

**Keywords:** covid‐19, preference, public contributors, public participation, remote

## Abstract

**Introduction:**

Covid‐19 expanded the use of remote working to engage with public contributors in health and social care research. These changes have the potential to limit the ability to participate in patient and public involvement and engagement (PPIE) for some public contributors. It is therefore important to understand public contributors' preferences, so that remote working can be organized in an optimal way to encourage rather than discourage participation.

**Methods:**

We use an economic preference elicitation tool, a discrete choice experiment (DCE), via an online survey, to estimate public contributors’ preferences for and trade‐offs between different features of remote meetings. The features were informed by previous research to include aspects of remote meetings that were relevant to public contributors and amenable to change by PPIE organizers.

**Results:**

We found that public contributors are more likely to participate in a PPIE project involving remote meetings if they are given feedback about participation; allowed to switch their camera off during meetings and step away if/when needed; were under 2.5 h long; organized during working hours, and are chaired by a moderator who can ensure that everyone contributes. Different combinations of these features can cause estimated project participation to range from 23% to 94%. When planning PPIE and engaging public contributors, we suggest that resources are focused on training moderators and ensuring public contributors receive meeting feedback.

**Discussion and Conclusion:**

Project resources should be allocated to maximize project participation. We provide recommendations for those who work in public involvement and organize meetings on how resources, such as time and financial support, should be allocated. These are based on the preferences of existing public contributors who have been involved in health and social care research.

**Patient or Public Contribution:**

We had a public contributor (Naheed Tahir) as a funded coapplicant on the UKRI ESRC application and involved members of the North West Coast Applied Research Collaboration (NWC ARC) Public Advisor Forum at every stage of the project. The survey design was informed from three focus groups held with NWC ARC public contributors. The survey was further edited and improved based on the results of six one‐to‐one meetings with public contributors.

## 1 INTRODUCTION

Covid‐19 prevention measures, which started in the United Kingdom in March 2020, forced a shift to remote forms of working in patient and public involvement and engagement (PPIE) in health and social care research. Due to shielding and social distancing, the usual ways of involving the public (such as face‐to‐face meetings and events) were not possible during the pandemic. Even though at the time of writing, Covid‐19 restrictions have largely been removed or substantially eased, remote working will continue to be used alongside face‐to‐face meetings and as part of ‘hybrid’ working. Remote working has provided a valuable way of continuing to do PPIE during the pandemic and ameliorating some of the isolation felt by public contributors during the periods of lockdown in 2020.^1^ However, remote working with its dependence on the Internet and communication equipment, needs to be carefully considered in light of socioeconomic and health inequalities. There is a digital divide that maps onto existing socioeconomic inequalities, with those in lower socioeconomic groups and older communities having less access to and opportunities to use remote working technologies.^2^ Areas of high deprivation and ethnic minority communities bear the burden of poor health and access to health care and these communities have experienced disproportional harmful effects of the pandemic.^3^, ^4^ As a consequence, health inequalities are increasing.^5^ Therefore, PPIE conducted remotely has the potential to further disenfranchise already disadvantaged groups and attention needs to be paid to ensuring diversity and inclusion in PPIE remote working.

The likelihood that remote working will continue alongside face‐to‐face meetings means that disenfranchisement due to the digital divide is added to concerns that PPIE was insufficiently diverse before the pandemic.^6^ A recent National Institute of Health & Care Research (NIHR) (a UK‐based funder of health and social care research) survey of public contributors found a lack of diversity in the public contributor community in terms of age and socioeconomic status and addressing this is an NIHR priority.^7^ This paper reports on a discrete choice experiment (DCE) survey that is part of a larger UK‐based study that explored remote working in PPIE in health and social care research during the Covid‐19 pandemic in 2020–2021.^8^

### 1.1 PPIE in health and social care research

PPIE has become a widespread phenomenon in health and social care research. The NIHR state: ‘Public involvement is at the centre of NIHR health and social care research, and the public has a right to have a say in what and how publicly funded research is undertaken’.^9^ The terms ‘patient and public involvement and engagement’ (PPIE) or public and patient involvement (PPI) are commonly used to capture a broad range of activities that aim to develop effective links between researchers and the general public. We will use a broad definition of PPIE for the purposes of this paper, ‘research being carried out ‘with’ or ‘by’ contributors of the public rather than ‘to’, ‘about’ or ‘for’ them.’ PPIE includes notions of active contribution,^10^ and ‘good’ PPIE is more about coproduction than just involvement.^11^ ‘Coproducing a research project is an approach in which researchers, practitioners and the public work together, sharing power and responsibility from the start to the end of the project, including the generation of knowledge’.^11^

We use the term ‘remote working’ to cover meetings and interactions held without face‐to‐face contact that use communication technologies such as telephones (landlines, mobiles, smartphones), computers, tablets, online conferencing/meetings software, social media, and apps. Hybrid meetings are where a meeting is held with some participants face‐to‐face and other participants joining remotely, such as via a video conferencing tool such as Zoom.^12^

There is limited research on the feasibility and assessment of remote working quality in PPIE. However, since the start of the Covid‐19 pandemic, an increasing number of guidelines and recommendations have been produced on how to undertake remote working in PPIE. These include: ‘Top tips for carrying out PPI activities during Covid‐19’ by the NIHR Research Design Service^13^; ‘How do I hold a PPI meeting using virtual tools?’ by the NIHR School for Primary Care Research^14^ and ‘Carry on coproducing: handy hints and tips to help you out’, by the University College London Public Engagement Blog.^15^ This paper contributes to this growing literature. In one of the few published articles, Lampa et al.^16^ reported observations of digital PPIE meetings during the pandemic. They found that meeting organizers need to be committed to solving practical issues and it is important to coproduce the meeting structure and format with public contributors. Adeyemi et al.^17^ discussed three case studies of remote PPIE with marginalized groups and concluded that it is possible to do remote work with such groups, but it also presents some challenges, predominately the challenge of digital poverty and lack of access to equipment and data/WIFI.

This study aims to provide evidence on what good practice in remote working in PPIE might look like. We used a DCE to elicit public contributors’ preferences for different features of remote communication and working, such as investment in technology, time commitment, training, and support needs. The DCE aimed to find out:

1. how much time and resources public contributors would be able and willing to invest in remote communication,
2. what features of remote communication are the most important to maximize public contributors’ participation, and
3. how public contributors trade off the different features when deciding whether to participate.

A better understanding of how to organize and support public contributors with remote working can help engage public contributors and allow teams to design remote working practices that are inclusive and encourage, rather than limit, diversity.

## 2 METHODS

DCEs are a survey‐based method grounded in economic theory that assumes the value of a service (in this instance PPIE meetings) comprises the value of the different attributes that describe it.^18^ DCEs are a widely used method to elicit preferences from the public, patients, and healthcare professionals.^19^ Respondents in a DCE are asked to make a series of choices between two or more hypothetical alternatives describing different types of meeting packages. These packages are further described by different features (herein referred to as attributes) and a corresponding value (herein referred to as levels). For example, a meeting attribute could be the ‘time of day the meeting takes place’ and the levels could be ‘between working hours’ and ‘between working hours and evenings’. When respondents make choices, they are implicitly trading the attributes and levels that describe the alternatives. This trade‐off information can be used to estimate the relative importance of one attribute over another and predict participation in a defined meeting package.

In this study, public contributors were asked to imagine they were invited to take part in a new project. This would mean that they had to join regular project meetings using video calls. In the DCE, public contributors were then presented with a series of choices. In each choice, they were shown two different ways in which project meetings could be organized. These meetings differed in seven attributes. Contributors were asked to choose to take part in one type of meeting or not take part in the project (e.g., opt‐out alternative). The DCE was designed using a state‐of‐practice sequential, mixed methods approach.^20^, ^21^

**Figure 1:**
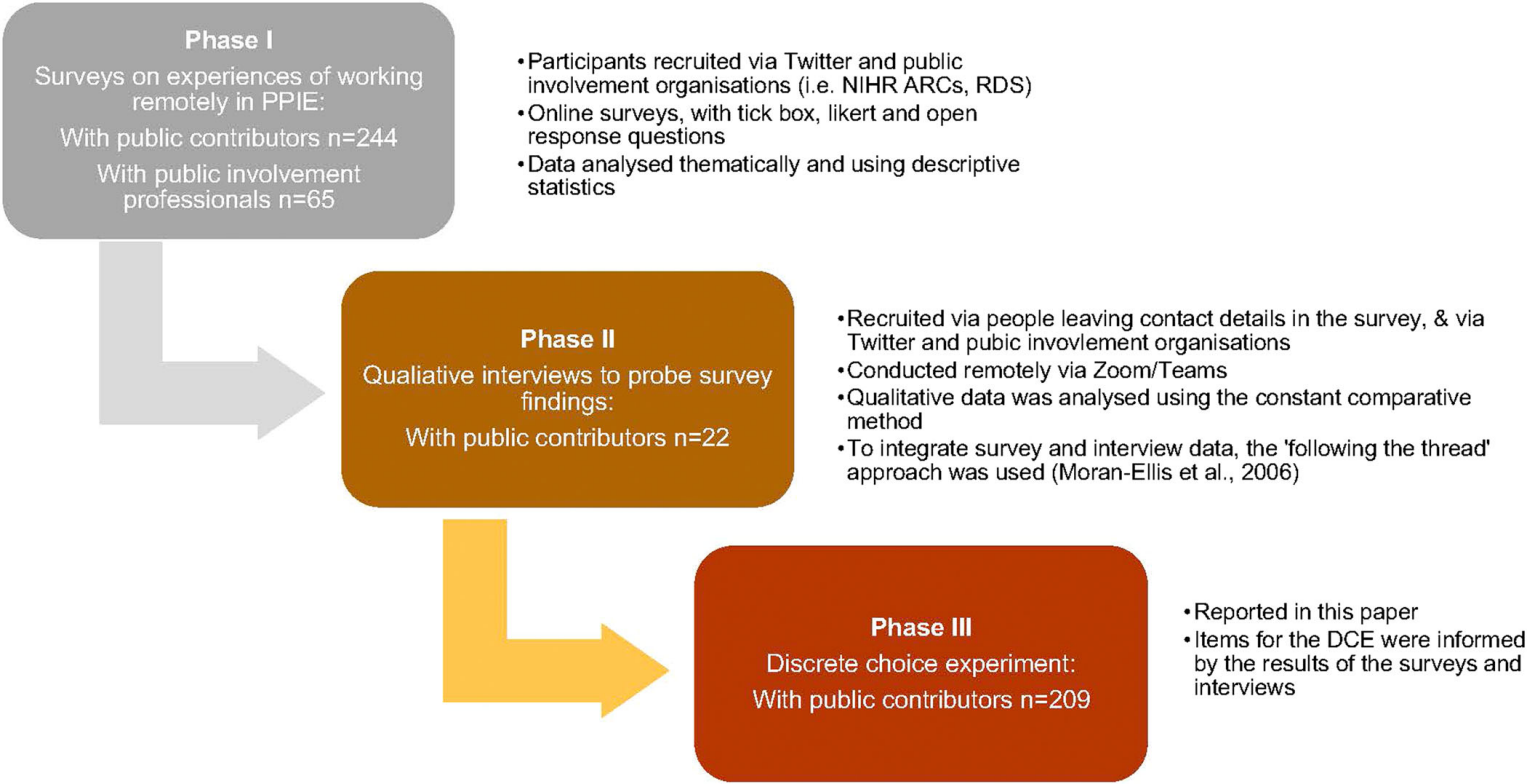
Flowchart of the remote working in PPIE study. PPIE, patient and public involvement and engagement.

The attributes and levels describing the remote meetings were identified and refined from the previous phases of this study (see Figure 1). This comprised two surveys, one with public contributors (*n* = 244) and one with those who worked in PPIE (*n* = 65) and subsequent qualitative interviews with public contributors (*n* = 22). The surveys asked general questions about the role and PPIE experience, digital literacy and different aspects of remote working. After analyzing the survey data, we conducted qualitative interviews to further probe and explore the themes (results of the previous phases are reported in Frith et al.^22^ and Jones et al.^23^). This ensured that the included attributes of remote meetings are those which are both most important to PPIE contributors and amenable to change or under the control of meeting organizers.

**Figure 2:**
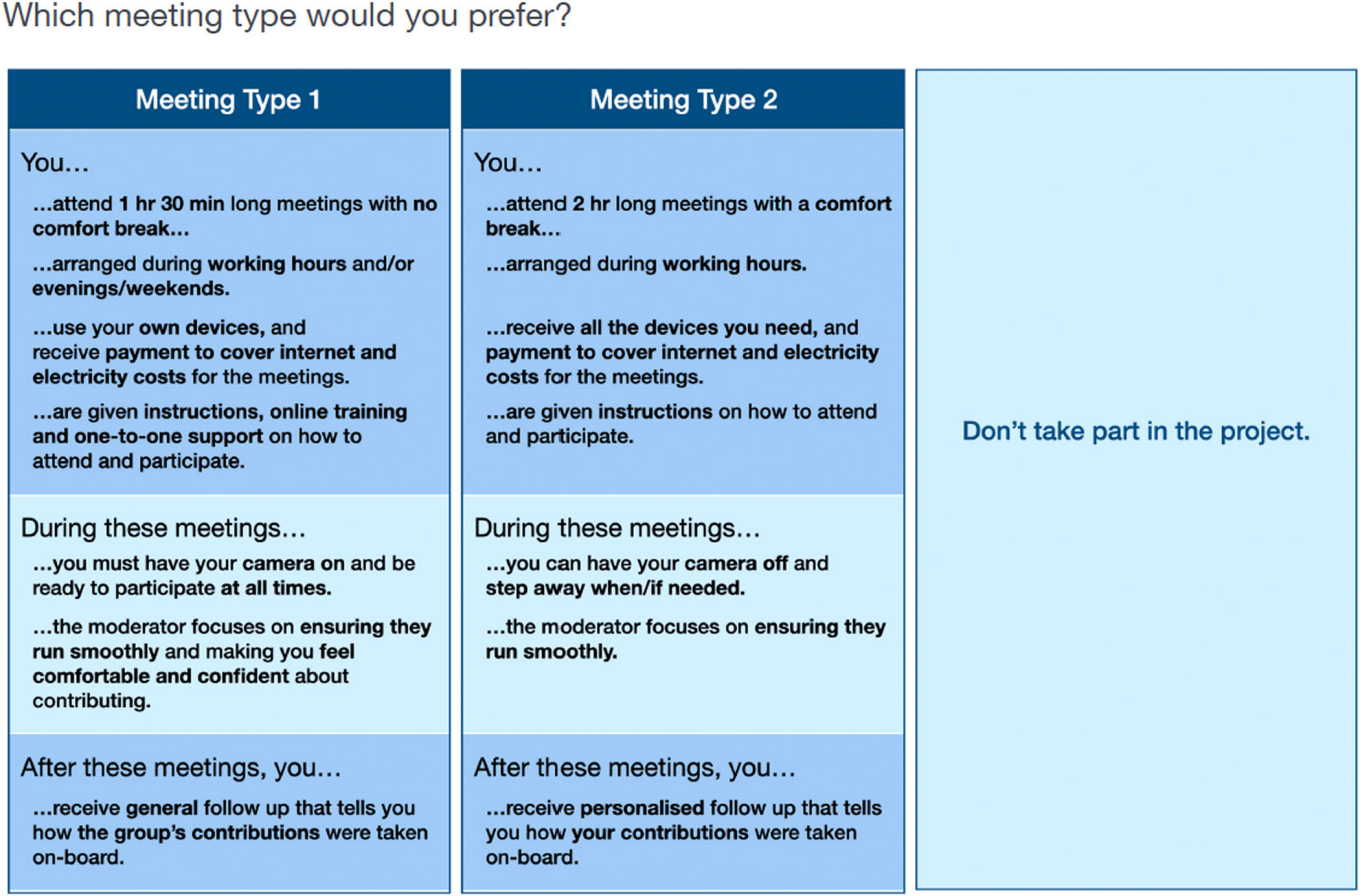
Example DCE choice task as seen by respondent. DCE, discrete choice experiment.

The data from the previous phases (Phase 1 and Phase 2) of our study identified three stages that influenced how PPIE contributors felt about participating in meetings: what happens before, during and after the meetings. Based on these findings, seven attributes, grouped into these three stages, were used to describe the remote meetings (see Figure 2). Four attributes described features of a meeting's organization that happen *before*: the length of the meeting, the time of day when the meeting is held, the type of connectivity support that is provided and the technical support provided to help participants to join and contribute during video calls. Two attributes described features that occur *during* meetings: the etiquette during a remote meeting and the role of the moderator. One attribute described whether any feedback on the contributors’ contributions was provided *after* the meeting. The rationale for the selection of these attributes and levels, based on the previous phases’ findings is described below (Table 1).

**Table 1:** Attributes and levels used in the choice experiment

Attribute	Levels
1. Length of meeting	1.5 h without comfort break.
	2 h with comfort break.
	2.5 h with comfort break and a socializing opportunity.
2. Time of day	Working hours.
	Working hours and/or Evenings/Weekends.
3. Connectivity	Use own device/Internet/electricity.
	Everything you need is provided (devices, Internet and electricity).
	Use own device. Internet and electricity expenses provided.
	Use own device and Internet. Electricity expenses provided.
4. Support during project (…on how to attend/participate)	Instructions only
	Instructions + online training
	Instructions + online training + one‐to‐one IT support
5. Etiquette during meetings	Expected to have camera on and be present throughout meeting.
	You can have camera off and you can step away when/if needed.
6. Role of moderator (Moderator focuses on…)	*(Standard moderator)* Only on ensuring meetings run smoothly.
	*(Great moderator)* On ensuring meetings run smoothly and makes an effort to make you feel comfortable and confident about contributing to meeting.
7. Meeting feedback (e.g., sense of contribution)	No follow up.
	General follow up that tells how broad contributions from the meeting were included.
	Personalized follow up that tells how individual contributions were included.

Length of the meeting was deemed a key feature that public contributors would want to know ahead of any remote meeting, with the data suggesting participants generally preferring shorter meetings, while some enjoyed meetings with an icebreaker and/or social activity that allowed them to interact with and get to know the other participants, albeit that is likely to lengthen the meeting time overall. Given the potential impersonal nature of remote meetings, we included the possibility of having a longer meeting with a social activity as one level in the DCE. The meeting's time of day was also important. Some contributors preferred the flexibility of meetings outside working hours and others preferred meetings during working hours, especially those with caring responsibilities. We include the provision of connectivity tools as an attribute that contributors would want to know before, as this allowed us to test whether providing web‐enabled devices and reimbursing Internet and electricity costs is a way to overcome the digital divide and increase participation. Similarly, we included the provision of technical support as this was identified to be a potential driver of public contributor disenfranchisement, especially amongst contributors with limited experience and Internet literacy.

Previous phases identified meeting etiquette as a potential key driver of meeting and project uptake. Public contributors in the interviews discussed the difficulty of balancing long video calls from home with their caring responsibilities, with some describing the difficulties they experienced when they have distractions at home. Some public contributors expressed a preference for having the flexibility to attend meetings anywhere and/or have the possibility to manage other things suddenly, such as attending to family members. The meeting etiquette attribute thus described whether contributors had to keep their cameras on and be ready to contribute during the whole meeting, or whether it was possible to turn them off and step away when needed. The role of the meeting's chair or moderator was another important aspect identified in the interviews and surveys in the previous phases (for our purposes we are using them interchangeably to mean ‘the person who is running the meeting’ or organizing the meeting, as these are not formal decision making meetings where the chair has a formal role). Contributors were able to distinguish between a good and a sub‐par moderator, with the majority agreeing on the importance this can have to the success of a meeting. We described this attribute in terms of a *standard moderator* who only ensures meetings run smoothly and a *good moderator* who also makes sure participants feel comfortable and confident to contribute.

A recurring theme in most of the interviews and survey data from the previous phases was the uncertainty of what happens after the meetings and, specifically, whether the public contributors had been listened to and their suggestions are taken on board. We, therefore, included the provision of feedback, either as a personalized report that details how each individual's contribution was used or a general report that explained how the group's contributions were taken onboard, as an attribute that can be both influenced by the meeting organizers and speaks to addressing this uncertainty. While not being an issue exclusive to remote meetings, this feature was deemed key given the nature of remote meetings and the way they can limit nonverbal communications between participants and moderators.

Based on the attributes and levels, there are 273,248 possible unique choice tasks (pairs of meeting descriptions). We used experimental design techniques to reduce these to a more manageable number. Specifically, we created a D‐efficient experiment design with vague informative priors and allowing for estimation of nonlinear effects of attributes using Ngene software to reduce the number of choice tasks to 24.^24^, ^25^ The aim of this design was to create realistic choice tasks with statistical properties that facilitate the estimation of the effect of each feature.^26^ To reduce respondent burden, the resulting design was blocked into three sets to each respondent was asked to complete eight choice questions.^27^ Based on this design, a minimum of 49 respondents were required for each analysis block to ensure the estimation of all attribute effects.^28^ Respondents were randomly assigned to one block and the order of the choice tasks within each block was also randomized to minimize ordering effects.^29^

The DCE online survey was comprised of three sections (see Supporting Information 1). Section 1 asked about respondents’ experience as PPIE contributors. Section 2 contained the attributes and levels. Section 3 included demographic questions to characterize the sample (age, number of children, self‐perceived health and education level). The survey was tested in *n* = 6 think‐aloud interviews with public contributors.

The DCE was administered as a self‐complete online survey of UK residents who had been involved in at least one PPIE project as a public contributor. Survey recruitment used a combination of a targeted and opportunistic sampling amongst existing UK PPIE networks and colleagues. Data were collected between 6 September and 1 November 2021. Participants were asked to provide informed consent before the start of the survey and participants were not given any financial incentives for completing the questionnaire. The participant information sheet provided participants with details on how to access the study results. The University of Liverpool, Institute of Population Health Ethics Committee granted ethical approval (REF: 7636).

### 2.1 Data analysis

#### 2.1.1 DCE analysis

The DCE response data indicates which one of the three alternatives a respondent selects in each choice task. The data were analysed using a mixed logit (MXL) model.^30^ We assume that respondents (*n*) choose the alternative (*j*) that provides them with the highest utility in each of the choice tasks (*t*). Following random utility theory,^31^ utility can be decomposed into a deterministic part, *V*, which is observable and based on the attributes included in the DCE, and a random component, *ε*, which is unobservable. The observable component is specified as a linear and additive function of the attributes and levels describing the meeting types, where

$$V_{\mathrm{njt}}=\beta_{0}{\mathrm{ASC}}_{\mathrm{opt}-\mathrm{in}}+\beta_{1}{\mathrm{length}}_{2\;h}+\beta_{2}{\mathrm{length}}_{2.5\;h}+\beta_{3}\mathrm{time}\;\mathrm{of}\;{\mathrm{day}}_{\mathrm{working}\;\mathrm{hours}\;and\;\mathrm{evenings}\;and\;\mathrm{weekends}}+\beta_{4}\mathrm{connectivity}\;{\mathrm{tools}}_{\mathrm{devices}\;and\;I\mathrm{nterne}t\;and\;\mathrm{electricity}\;\mathrm{costs}}+\beta_{5}\mathrm{connectivity}\;{\mathrm{tools}}_{I\mathrm{nternet}\;and\;\mathrm{electricit}y\;\mathrm{costs}}+\beta_{6}\mathrm{connectivity}\;{\mathrm{tools}}_{\mathrm{electricity}\;\mathrm{costs}}+\beta_{7}\mathrm{tech}\;{\mathrm{support}}_{\mathrm{training}\;\mathrm{videos}\;and\;1\;\mathrm{to}\;1\;\mathrm{support}}+\beta_{8}\mathrm{tech}\;{\mathrm{support}}_{\mathrm{training}\;\mathrm{videos}}+\beta_{9}{\mathrm{etiquette}}_{\mathrm{camera}\;\mathrm{off}\;and\;\mathrm{ca}n\;\mathrm{step}\;\mathrm{away}}+\beta_{10}{\mathrm{moderator}}_{\mathrm{good}}+\beta_{11}{\mathrm{followup}}_{\mathrm{general}}+\beta_{12}{\mathrm{followup}}_{\mathrm{personalised}}$$

The *ASC_opt‐in_* is an Alternative Specific Constant which takes a value of one for alternatives which have the participant opting into the project. This can be interpreted as the general preference to choose to take part in a project with remote meetings compared to opting out. The *β* parameters reflect the observed change caused by each of the meeting attributes/levels to the overall utility (e.g., benefit) derived from taking part in a project involving remote meetings. To allow for preferences to vary across the sample, the *β*_1_ *–β*_12_ parameters are assumed to be normally distributed. We estimate the mean and a standard deviation for each parameter, where the latter's statistically significant would indicate if the attribute's preference varied across the sample. Positive mean coefficients represent increases in utility, and negative coefficients as a loss in utility from the corresponding base level, which can be interpreted as whether they increase or decrease the likelihood of choosing to take part in a project. *β*_1_–*β*_12_ attributes are effects coded, thus allowing the postestimation of mean estimates for all attribute levels.^32^ The model is estimated using simulated maximum likelihood with 500 Halton Draws.

We then use the parameters to estimate participation in different remote meeting configurations. Participation probability for different scenarios *h* is estimated using

$$P(x_{h},\beta_{k})=\frac{\exp\left(\beta_{0}+\sum_{k\in \left[1,12\right]}\beta_{k}x_{hk}\right)}{\sum_{j\in \left[1,2\right]}\exp\left(\beta_{0}+\sum_{k\in \left[1,12\right]}\beta_{k}x_{hk}\right)},$$

where *β* denotes the parameter of attribute *k* and *x*_*jk*_ is the level that the attribute takes in the scenario *h*.

We estimate the participation of different remote meeting configurators described in Table 2. For example, Scenario 1 describes meetings that are 2 h long, organized during working hours, where contributors can step away if needed, with a good moderator who provides general feedback. Scenario 4 describes a similar meeting organization but is less resource intensive as it does not provide any feedback. By comparing Scenarios 1 and 4, it is possible to calculate the effect of providing general feedback on participation. The chosen configurations in Table 2 describe different ways a meeting can be organized, and each involves a different allocation of resources available to meeting organizers. All analysis was done using the statistical software R. Confidence intervals were computed using the delta method.

**Table 2:** Features for scenarios used in participation rate analysis

Scenario	Length	Time of day	Etiquette	Moderator	Feedback
1	2 h with break	Working hours	Camera off and can step away	Run smoothly and confident participation	General feedback
2	2 h with break	Working hours	Camera on and ready at all times	Run smoothly and confident participation	Personalized feedback
3	1.5 h	Working hours and weekends/evenings	Camera on and ready at all times	Only ensures meetings run smoothly	General feedback
4	2 h with break	Working hours	Camera off and can step away	Run smoothly and confident participation	No feedback
5	1.5 h	Working hours and weekends/evenings	Camera off and can step away	Only ensures meetings run smoothly	No feedback
6	2.5 h with break and activity	Working hours	Camera off and can step away	Run smoothly and confident participation	No feedback
7	1.5 h	Working hours and weekends/evenings	Camera on and ready at all times	Only ensures meetings run smoothly	No feedback
8	2.5 h with break and activity	Working hours and weekends/evenings	Camera on and ready at all times	Only ensures meetings run smoothly	No feedback

#### 2.1.2 Affordance theory

We drew on the concept of affordances to further analyse our data. The features of remote meetings can be conceptualized as furthering particular affordances. Building on Gibson's work, Norman defines affordance as ‘the relationship between a physical object and a person…. [the] relationship between the properties of an object and the capabilities of the agent that determine just how the object could be possibly used’.^33^ He gives the example of a chair, a chair affords—is for—sitting. There are many different potential affordances when actors use an object or artefact (such as remote meetings), there are ‘bundles’ of affordances. These bundles are not independent but interact, and this was captured by the attributes and levels used in this DCE.

## 3 RESULTS

Two‐hundred and nine respondents completed the survey. Respondents were evenly split across the three analysis block sets of eight‐choice questions. The median completion time was 14 min 29 s. The sample characteristics are described in Table 3. The modal respondent was an experienced PPIE contributor (e.g., involved in three PPIE projects), had taken part in remote meetings as part of their role, had access to devices and an Internet connection to enable joining remote meetings and was able to take part in remote meetings uninterrupted. Only 22% of respondents were completely certain that past contributions to other projects had been taken onboard (64% were at least very certain). Respondents were more likely to be female, over the age of 45, highly educated (at least University or equivalent), living alone or with no more than one person, and having no caring responsibilities.

**Table 3:** Characteristics of respondents

*Sociodemographic characteristics*			
Age			
18–24	1		0.5%
25–34	5		2.4%
35–44	10		4.8%
45–54	28		13.4%
55–64	55		26.3%
65+	109		52.2%
Prefer not to say	1		0.4%
Sex			
Female	133		63.6%
Male	76		36.4%
…Is it the same as gender you identify with			
Yes	193		92.3%
No	2		1.0%
Prefer not to say	14		6.7%
Ethnicity			
White	185		88.5%
Mixed or multiple ethnic groups	6		2.9%
Asian or Asian British	4		1.9%
Black, Black British, Caribbean or African	5		2.4%
Other	5		2.4%
Prefer not to say	4		1.9%
Marital status			
Single	40		19.1%
Married, civil partnership or cohabiting	126		60.3%
Separated	2		1.0%
Divorced	11		5.3%
Widowed	25		12.0%
Prefer not to say	5		2.4%
Caring responsibilities			
Yes	68		32.5%
No	139		66.5%
Prefer not to say	2		1.0%
Highest level of education			
No qualifications	6		2.9%
GCSE or equivalent	17		8.1%
A levels or equivalent	20		9.6%
Apprenticeship or equivalent	33		15.8%
University or equivalent	125		59.8%
Other	8		3.8%
English first language			
Yes	202		96.7%
No	7		3.3%
Employment			
Full time employment	29		13.9%
Part time employment	21		10.0%
Retired	110		52.6%
Student	6		2.9%
Carer	8		3.8%
Unemployed	3		1.4%
Adults in household			
1	55		21.9%
2	119		47.4%
3	27		10.8%
4	7		2.8%
More than 4	1		0.4%
Children in household			
0	190		90.9%
1	13		6.2%
2	5		2.4%
3	1		0.5%
More than 3	0		0.0%
Household income			
£0–£10,400	20		9.6%
£10,400–£20,800	31		14.8%
£20,800–£31,200	47		22.5%
£31,200–£52,000	34		16.3%
£5200–	30		14.4%
Prefer not to say	47		22.5%
*Experience as a contributor*			
Involved in how many projects?			
None	9	4.3%	
One	26	12.4%	
Two	38	18.2%	
Three	36	17.2%	
Four	11	5.3%	
More than four	89	42.6%	
…Out of the those involved in at least one:			
Is any project doing remote meetings?			
Yes	190	95.0%	
No	10	5.0%	
Involved in how many organizations as PPIE advisor			
One	79	37.8%	
Two	70	33.5%	
Three	43	20.6%	
More than 3	17	8.1%	
…which organizations?			
National Institute of Health Research (NIHR) organization or other government‐funded research (MRC, ESRC, etc.)	166	79.4%	
Third sector organization or charity (e.g., Alzheimer's Society, Cancer Research)	81	38.8%	
The NHS or social care organization (e.g., a hospital trust, Clinical Commissioning Group, local authority)	123	58.9%	
Other	46	22.0%	
Involved in what capacity			
Carer	57	27.3%	
Patient/service user	171	81.8%	
Member of public/neighbourhood/community	128	61.2%	
Other	21	10.0%	
Currently has access to:			
Computer/laptop with webcam	191	91.4%	
Tablet (or iPad)	110	52.6%	
Mobile phone with camera	141	67.5%	
Stable Internet connection (home broadband or mobile network)	179	85.6%	
Headset/headphones with microphone	79	37.8%	
In the past, has received payment to cover:			
Internet access	50	23.9%	
Electricity bills	15	7.2%	
Is able to take part in video calls without interruptions			
Yes	175	83.7%	
Certainty past contributions have been taken on board			
Not at all certain	10	4.8%	
Somewhat certain	20	9.6%	
Moderately certain	45	21.5%	
Very certain	88	42.1%	
Completely certain	46	22.0%	
Agree with given definition of a great moderator			
Strongly disagree	21	10.0%	
Somewhat disagree	6	2.9%	
Neither agree nor disagree	9	4.3%	
Somewhat agree	45	21.5%	
Strongly agree	128	61.2%	

We found most respondents were willing to take part in a project that involved remote meetings and were willing to make trade‐offs across the remote meeting attributes (in the DCE, *Meeting Package A* [first displayed alternative], *Meeting Package B* [second displayed] and *Not taking part* [third displayed] were chosen 43.8%, 47.0% and 9.2% of the time, respectively. Three respondents [1.9% of the sample] always chose the ‘not to take part’ option in all choice tasks). The DCE results are shown in Table 4. The ASC has a statistically significant positive parameter which suggests, on average, respondents are more likely to take part in a project involving remote meetings compared to not taking part. Statistically significant parameter estimates indicate that respondents prefer meetings which: are shorter (less than 2.5 h without a social activity); scheduled during working hours; permit them to have their cameras off and step away if needed; have a moderator that ensures participants are comfortable and confident, and provide feedback about how contributions were taken on board. There was no statistical difference between types of feedback, which suggests respondents do not distinguish between general or personalized feedback. The provision of devices or reimbursement of costs and receiving additional support such as training videos or one‐to‐one support to connect to meetings were not statistically significant and thus did not have an effect on the likelihood that respondents chose a type of meeting package.

**Table 4:** Parameter results from DCE choice questions

	Mean		Standard deviation	
Attribute	Estimate.	*p* Value	Estimate	*p* Value
Alternative Specific Constants (ASC)				
Opting in (e.g., choosing a meeting type)	1.140	<.001	‐	‐
Length				
1.5 h with no comfort break	0.247	.006	‐	‐
2 h with comfort break	0.381	<.001	0.250	.056
2.5 h with comfort break and social activity	−0.628	<.001	0.801	<.001
Time of day				
Working hours only	0.160	.008	‐	‐
Working hours, weekends and evenings	−0.160	.008	0.526	<.001
Connectivity tools (provide with…)				
Nothing	−0.084	.389	‐	‐
Devices, Internet and electricity costs	−0.064	.459	0.073	.883
Internet and electricity costs	0.042	.617	0.219	.268
Electricity costs	0.106	.256	0.104	.711
Support to connect (provide with…)				
Instructions	0.008	.907	‐	‐
Instructions and training videos	−0.051	.509	0.134	.504
Instructions and training videos and one‐to‐one support	0.043	.548	0.046	.815
Remote meeting etiquette				
Camera on and ready to take part	−0.390	<.001	‐	‐
Camera off and can step away when/if needed	0.390	<.001	0.677	<.001
Moderator				
Ensures meetings run smoothly	−0.091	.084	‐	‐
Also ensure comfortable and confident about contributing	0.091	.084	0.091	.493
Follow up (i.e., sense of contribution)				
No follow up	−1.058	<.001	‐	‐
General follow up document	0.565	<.001	0.785	<.001
Personalized follow up document	0.494	<.001	0.389	.033

*Note*: Log‐likelihood = −1355.708. Number of observations = 1672. Akaike information criterion = 2761.415.

Abbreviation: DCE, discrete choice experiment.

**Figure 3:**
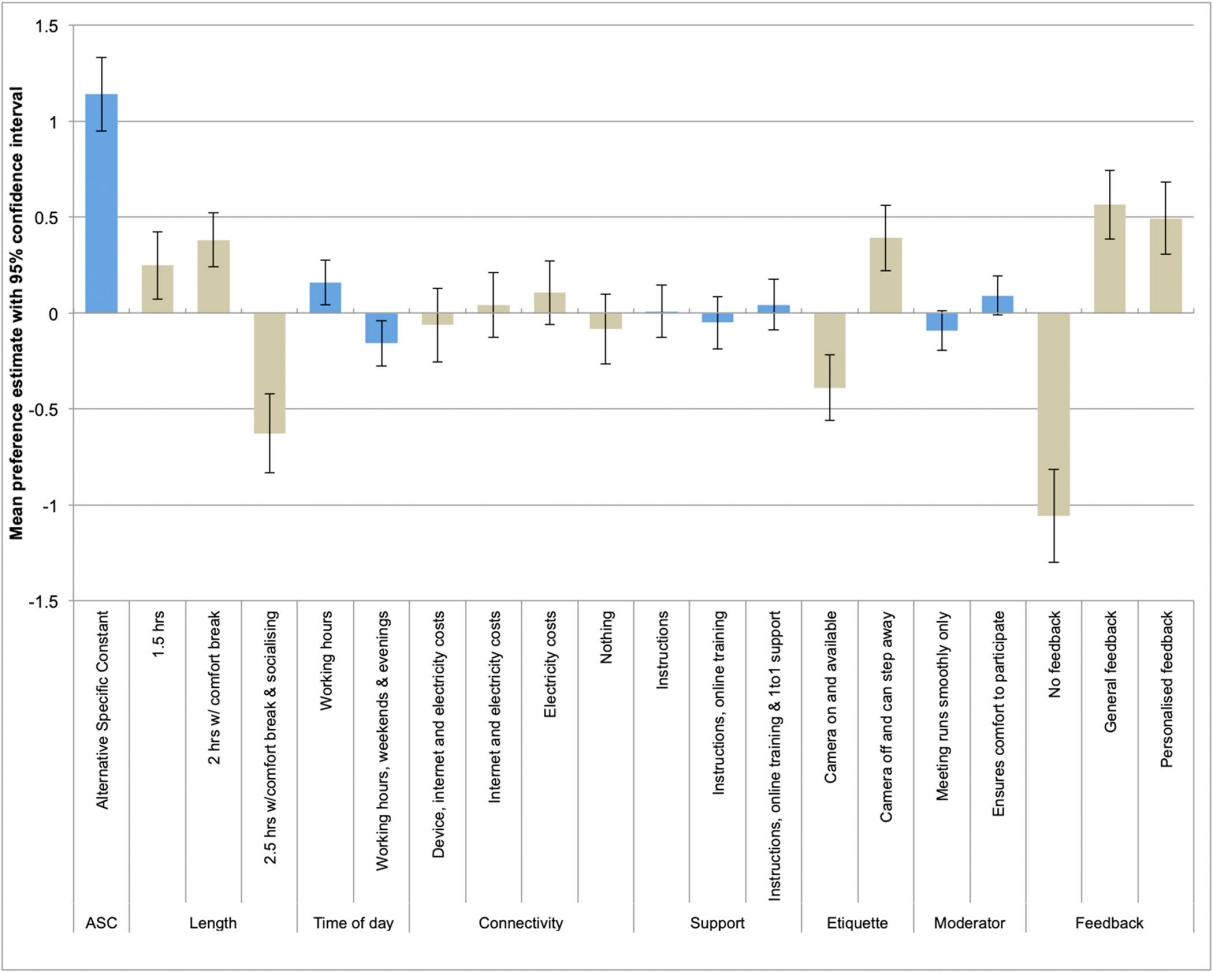
Contribution of parameter estimates to utility

Figure 3 shows the contributions to the overall utility and illustrates the trade‐offs between meeting features respondents were willing to make. For example, the positive effect on the participation of being able to have the camera off and step away from the meeting if needed is not statistically different from the negative effect of having a 2.5‐h long meeting. This suggests respondents could be compensated for taking part in longer meetings as long as they are able to have their cameras off. Similarly, meetings with a great moderator/chair can compensate for meetings that are organized outside working hours (e.g., weekends and evenings).

**Figure 4:**
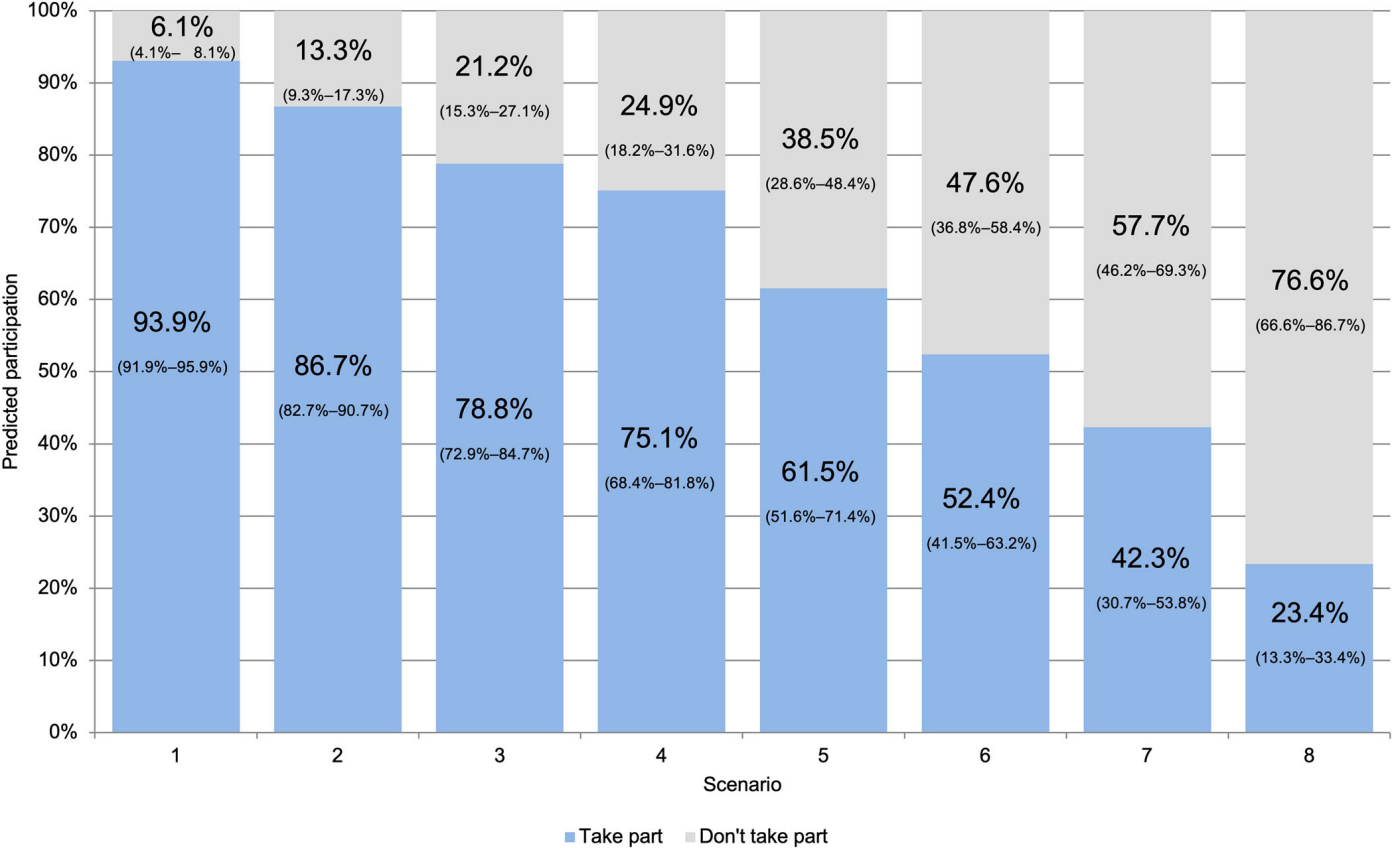
Participation in different meeting configuration scenarios

Figure 4 shows the estimated participation for the different meeting configurations. Potential participation ranges from 23% to 94% depending on the remote meeting features. A project with the most desirable meeting features (e.g., Scenario 1: meetings that take 2 h with a comfort break, organized during working hours, with a great moderator, where participants can step away if needed and for which they received personalized feedback) has an estimated participation rate of 94%. Conversely, a project with the least desirable features (e.g., Scenario 8: meetings that take 2.5‐h, organized outside working hours, with a standard moderator, where participants are expected to have the camera on and ready to contribute at all times, and for which they receive no feedback) would have a predicted participation of 23%.

Overall, the biggest effect on participation is the provision of feedback, followed by the length of the meeting (with shorter meetings being preferred) and whether respondents can turn their cameras off can step away if needed. For example, in a meeting with all the most desirable features, except the provision of feedback (Scenario 4) participation would be reduced by 19% (from 94% to 75%). As expected, features with nonstatistically significant parameters (e.g., providing devices and costs or extra support to connect to meetings) had a limited effect on participation and were not considered in the scenario analysis.

## 4 DISCUSSION

To our knowledge, this is the first DCE to investigate public contributors’ preferences for how remote meetings in PPIE are organized. While remote working has the potential to limit the ability to participate for some public contributors, for others it can increase their participation.^23^ People in our sample were generally willing to take part in projects even if this involves remote meetings. However, project participation can vary significantly depending on certain features of the meetings. Our findings suggest how project resources, such as time and financial support, can be best allocated to increase meeting participation by public contributors. Giving participants feedback about how their contributions to meetings are taken onboard by the organizers—how their contribution has made a difference was important to our participants. This has been found in other research in this area^34^ and providing feedback to public contributors has been described as an important, but often overlooked, part of PPIE leads’ work.^35^ Respondents seemed indifferent to whether this is general or personalized feedback, so there is little benefit from the additional resource cost of providing individual reports compared to a general one. We are not able to conclude if the importance of providing feedback is exclusive to remote meeting settings. However, our data suggest that this feature is important regardless of whether the meeting is remote or face‐to‐face. Furthermore, most respondents in our survey stated that they were not certain that their contributions had been taken on board in past projects. Given remote meeting settings can limit the nonverbal interactions and communication between all members of the team, it is likely that providing feedback that directly signals how their contributions were taken onboard is even more important in remote working.

We found that a meeting feature that is not resource intensive such as having remote meeting etiquette that permits participants to have their cameras off and step away is very important for increasing project participation. It is likely people value the flexibility to attend to other things, such as caring responsibilities while taking part in meetings. While having a moderator who ensures participants are comfortable to contribute was deemed less important, it is probable that resources should still be invested towards training or having experienced moderators/chairs. In the context of remote meetings where people might not be able to take part at all times, the role of the moderator to ensure that such flexible approaches result in meetings that run smoothly is key. Finally, we also found that long remote meetings should be avoided. Contributors are willing to forego the inclusion of social activities if the meetings are shorter. Resources allocated to arranging longer meetings with social activities should rather be focused on other features, such as moderator training and/or the provision of some type of postmeeting feedback to participants.

The features of remote meetings can be conceptualized as furthering particular affordances. Affordance theory has been used extensively in information technology and information systems research, to theorize the relationships between people and digital technologies. Thus, this theory is useful for understanding how public contributors made use of and interacted with remote working technologies. Volkoff and Strong^36^ apply affordance theory to information systems research, for them, ‘The power of the Affordance lens is that it helps to pinpoint the actors involved and the variety of potential actions they might engage in as they use the technology’ (p. 5).

This DCE experiment shows the relative importance of the different means of bringing about, what has been found to be, key affordances in remote working. From our data, we developed three affordances: Affordance 1: reducing the burden of remote meetings; Affordance 2: involving everyone in the meeting; Affordance 3: influencing and improving research. For example, an important affordance for public contributors was ‘making remote meetings less burdensome’. This DCE showed which features, such as length of meeting, camera use, and time of day of meetings were most important to our participants in terms of furthering this affordance (see Table 5). Bringing an affordance lens to our data enabled us to see how different features and elements of remote meetings interacted to understand how these different features afforded specific types of benefits to public contributors.^37^

**Table 5:** Elements giving rise to an affordance

Remote meeting features	Characteristics of actors
*Affordance 1: Reducing the burden of remote meetings*	
Camera	Public contributors can have their camera on or off
Length of meeting	Public contributors know they have time for meetings
Flexibility of attendance	Ability to step away, makes meetings less intense, public contributors can‐do other things if needed
During working hours	Convenient for public contributors
*Affordance 2: Involving everyone in the meeting*	
Moderator	Everyone is given an opportunity to be involved
Etiquette	Everyone knows how to get involved
*Affordance 3: Influencing and improving research*	
Feedback	Public contributors feel valued and that their contribution is important

*Source*: Adapted from Strong et al.^37^

### 4.1 Strengths and limitations

The strength of this study is how we generated the attributes and levels to include in the DCE. This DCE was nested in a larger study that included two surveys, with public contributors and public involvement professionals, qualitative interviews and focus groups with public contributors. Therefore, the attributes and levels that were used had a firm evidence base. There are two limitations of our study. Firstly, our online survey administration will have impacted on the sample size and composition. The ongoing Covid‐19 pandemic meant that we had to focus on online data collection and use existing contributor networks to distribute the survey link. We aimed to produce a short and well‐presented survey that was easily and widely accessible so that it minimized data attrition and respondent drop‐out rates, but future research could explore other sampling strategies. The Covid‐19 pandemic also meant that many ongoing projects were using remote meetings, and therefore many people in existing contributor networks are now experienced in joining remote meetings. This may explain why we found, on average, that providing contributors with connectivity tools (e.g., devices or covering costs) and ongoing technical support to connect to meetings had no impact on project participation. Second, respondents had both technological literacy skills and experience. Ideally, we would have compared the preferences of experienced and inexperienced respondents. We did not have enough respondents who were inexperienced or had low technological literacy skills to perform subgroup analysis. This means that we cannot explore how a digital divide may affect preferences, not least as the survey was completed online. In the case of technological support to connect to meetings, while it is likely that if the public contributor has no experience some training/support is needed at the beginning of the project, our results show that once the person gains experience there is no need to allocate resources to provide ongoing support or training.

## 5 CONCLUSION

Our results provide important insights for researchers involved in the design and organization of meetings that include public contributors. The shift to remote meetings with public contributors caused by Covid‐19 is likely to become a feature of PPIE. It is key we understand preferences and key drivers of project uptake to ensure remote meetings are designed so that potential public contributors are not disenfranchised. Hybrid meetings are also becoming popular, and further research is needed on these types of meetings, as public contributors’ preferences may be different in a hybrid meeting format, than when working solely online. We found that particular features of remote meetings can have a significant impact on project uptake, in our case ranging from 23% to 93% uptake. We identified features such as the provision of feedback, the role of the moderator, whether contributors need to have their cameras off and can step away, and whether the meeting length can have an impact on potential project uptake. We also found that features such as the provision of connectivity tools and support to connect to meetings did not have a significant effect, although this could be due to our sample having significant experience in remote meetings. Resources would be best allocated to moderator training and the provision of postmeeting feedback instead of arranging long meetings with socializing activities and providing ongoing technical support. These findings are useful for researchers, project managers and PPIE leads to inform the allocation of resources when designing remote meetings with public contributors. An allocation of resources that responds to contributors’ preferences will likely result in higher uptake of public involvement in projects.

## AUTHOR CONTRIBUTIONS

Shaima Hassan, Mark Gabbay, Naheed Tahir, Muhammad Hossain, Mark Goodall and Lucy Frith were involved in the conception of the study. Luis E. Loria‐Rebolledo, Verity Watson, Shaima Hassan, Mark Gabbay, Naheed Tahir, Muhammad Hossain, Mark Goodall and Lucy Frith were involved in the design of the study. Muhammad Hossain, Shaima Hassan, Lucy Frith carried out the focus group discussions with public contributors, and Lucy Frith, Muhammad Hossain and Shaima Hassan performed the qualitative analyses. Luis E. Loria‐Rebolledo carried out the survey development think‐aloud interviews and led the quantitative analysis. Verity Watson reviewed the statistical model and was involved in the data analysis. Luis E. Loria‐Rebolledo, Verity Watson, Shaima Hassan and Lucy Frith were involved in the original draft preparation. All authors helped shape the overall interpretation of the findings, and critically revised, edited and approved the final version of the manuscript.

## CONFLICT OF INTEREST

The authors declare no conflict of interest.

## Supporting information

Supporting information.

## Data Availability

The data that support the findings of this study are available from the corresponding author upon reasonable request.
