# Supplementary material for: Public contributors' preferences for the organization of remote public involvement meetings in health and social care: A discrete choice experiment study
Source: Health Expect. 2022 Nov 6;26(1):146–59. doi: 10.1111/hex.13641 (PMC9854307; doi:10.1111/hex.13641)
Supplement: Supplementary file 1 — Supporting information. [file HEX-26--s001.pdf]

Intro + Screening

Remote Patient Public Involvement and Engagement (PPIE)

You have been asked to take part because you **have been or are a public contributor**, and/or have an affiliation or an **association with an organisation that organises**, carries out or is involved with health and social care research, as a **public contributor/advisor**.

This survey is part of the Remote Working in Public Patient Involvement and Engagement (PPIE) project funded by the UKRI ESRC, that aims to investigate and improve ways of

doing PPIE remotely in health and social care research.

> Public - we include patients, potential patients, carers and people who use health and social care services as well as people from organisations that represent people who use services.
>
> Involvement – where members of the public are involved in research and/or organisations.
>
> Engagement – where information and knowledge about research is provided and disseminated.

This survey will ask you about different aspects of how remote meetings can be organised, including a section in which you will be asked to compare different meeting types and choose the one you prefer.

For more information on public involvement see: NIHR Centre for engagement and dissemination
https://www.nihr.ac.uk/documents/ppi-patient-and-public-involvement-resources-for-applicants-to-nihr-research-programmes/23437

Have you been or are you involved in patient public involvement and engagement (PPIE) as a public contributor?

Yes No

Screened out

Unfortunately you don't meet the criteria to take part in this study. Thank you for your interest.

Click **Next** to end the survey.

Screened in

Welcome.

Study Title: Exploring remote working practices for patient public involvement and engagement (PPIE) in health and social care research – Responding to COVID-19

Version number & date: 1.4. 22nd July 2021
Research ethics application number: 7363

You are being invited to participate in this research project. Before you decide whether to participate, it is important for you to understand why the research is being done and what it will involve.

Please take time to read the following information carefully and feel free to ask us if you

would like more information or if there is anything that you do not understand. Please also feel free to discuss this with your colleagues, friends or relatives if you wish.

We would like to stress that you do not have to accept this invitation and should only agree to take part if you want to.

You do not have to take part, participating is entirely voluntary.

Thank you for reading this.

For more information please see the Patient Information Sheet.

Click **Next** to continue.

About the study...

Below you will find information about the study and survey.

Why are we doing this study?
This project is part of the National Institute for Health Research (NIHR) Applied Research Collaboration North West Coast portfolio of research and funded by the UKRI ESRC Rapid Covid call.
The aim of the project is to explore how we can facilitate and improve doing patient public

involvement and engagement (PPIE) remotely, working with people without face-to-face contact, in health and social care research.

What will happen if I take part?
You have been invited to participate in this online survey. This survey will be made up of 33 questions asking what you think about remote public involvement meetings (i.e. online via video conference or other remote technologies). The survey should only take about 15-20 minutes to complete.

How will my data be used?
The University processes personal data as part of its research and teaching activities in accordance with the lawful basis of 'public task', and in accordance with the University's purpose of "advancing education, learning and research for the public benefit".
For more information please see the Patient Information Sheet.

There will be no payment for taking part in the study.

We do not think that there are any risks to taking part. If any of the issues discussed are distressing, you are free to close the survey. You do not have to answer any questions that you feel uncomfortable with.

Are there any benefits in taking part?

There are no direct benefits to you as an individual participating in this project, but this project will hopefully improve the conduct of remote working in PPIE and help make PPIE activities more accessible.

What will happen to the results of the study?
We will use the data to improve practice in remote working in PPIE. We will produce guidelines, reports, academic journal articles and present our findings at events and conferences. Reports and guidance will be open access and hosted on the ARC's website where you can access them if you are interested https://arc-nwc.nihr.ac.uk/

What will happen if I want to stop taking part?
You can stop taking part in the survey at any time and not submit the survey.

What if I am unhappy or if there is a problem?
If you are unhappy, or if there is a problem, please feel free to let us know by contacting Lucy Frith, L.J.Frith@liverpool.ac.uk, 0151 795 5333, and we will try to help.

Who can I contact if I have further questions?
The Remote PPI Project Team
rwppi@liverpool.ac.uk

Click **Next** to continue.

Participant Consent Form

By submitting this form, you are indicating that you have read the description of the study, are over the age of 18, and that you agree to the terms as described.

I confirm that I have read and have understood the information sheet dated 22nd July 2021 for the above study, or it has been read to me. I have had the opportunity to consider the information, ask questions and have had these answered satisfactorily.	Agree
I understand that taking part in the study involves answering survey questions about my PPIE role and public contributors being involved in working remotely in PPIE in health and social care research.	Agree
I understand that my participation is voluntary and that I am free to stop taking part and can stop completing the survey at any time without giving any reason and without my rights being affected.	Agree
I understand I can stop taking part in the survey at any time and close the browser, any data that has already been entered will be used. Once you entered any data into the survey you cannot withdraw your data as the survey answers are anonymous.	Agree
I understand that the information I provide will be held securely and in line with data protection requirements at the University of Liverpool.	Agree
I understand that my survey responses will be anonymous and only relevant and limited personal information will be collected about me.	Agree
I understand that my responses will be kept strictly confidential. I give permission for members of the research team to have access to my fully anonymised responses and that the anonymised data be made available to other researchers.	Agree
I understand and agree that any written comments will be used for the following purposes: anonymised written quotes to be used in publications and reports.	Agree

I agree to take part in the above study.

Yes No

Not consented

Thank you for your interest in the study.

Click **Next** to end the survey.

About your role

About your role...

We would like to ask you about your role as a public contributor/advisor to research projects in the past and at the moment.

For which of the organisations below have you been a public contributor/advisor on a research grant or project? *Please select all that apply.*

National Institute of Health Research (NIHR) organisation or other government-funded research (MRC, ESRC, etc.)

Third sector organisation or charity (e.g. Alzheimer's Society, Cancer Research)

The NHS or social care organisation (e.g. a hospital trust, Clinical Commissioning Group, local authority)

Other

If you selected 'Other', please specify:

How long have you been a public contributor/advisor?

0-6 months

6-12 months

1-2 years

2-3 years

4-5 years

More than 5 years

Are you a public contributor/advisor in any of the following capacities? *Please select all that apply*

As a carer

As a patient/service user

As a member of the public/neighbourhood/community

Any other

If you selected 'Other', please specify:

How many projects are you involved in as a public contributor/advisor at present?

None

One

Two

Three

Four

More than four

Are any of those projects currently holding remote meetings using video or telephone calls?

Yes	No

Attribute Intro

About the project meetings...

Being involved in a project as a public contributor/advisor involves attending a number of project meetings.

The COVID-19 pandemic has caused some of these meetings to be held remotely using video calls. It is possible that in the future remote meetings will become frequent after the pandemic.

The next questions will ask about your views on how remote meetings should be organised.

Given a choice, which of the following meeting lengths would you prefer for a regular remote video call project meeting?

1 hr 30 mins with no comfort break

2 hr with a comfort break in the middle

2 hr 30 min with a comfort break in the middle and a social activity.

What time of day would you prefer remote video call project meetings to take place?

During working hours (9am to 5pm)

During evenings/weekends

During working hours and/or evenings/weekends.

Do you have access to any of the following remote communication tools to connect to project meetings carried out as video calls? *Please select all that apply.*

Computer/Laptop with webcam

Tablet (or iPad)

Mobile phone with camera

Stable internet connection (home broadband or mobile network)

Headset/headphones with microphone

In previous projects, have you received any of the following payments to help cover the following costs of attending remote meetings?

Internet access in your home (e.g. broadband) and/or data plan for your mobile/tablet	Yes	No
Electricity bills	Yes	No

Thinking about your living arrangements and personal commitments (including caring responsibilities), are you able to take part in video call meetings that require you to have your webcam on at all times and without interruptions?

Yes

No

Project meetings usually are led by a moderator (or a facilitator) who ensures that meetings run smoothly.

Moderators can also take on other roles. Please state your level of agreement to the following statement:

"*A great moderator should* ***also*** *foster a social feeling through comments and language designed to make participants feel comfortable and confident, and foster a feeling of community among participants as they engage with each other.*"

Strongly disagree

Somewhat disagree

Neither agree nor disagree

Somewhat agree

Strongly agree

Thinking about the most recent project you have been/are involved in, how certain are you that your contributions during meetings have been taken on-board and impacted the project?

Not at all certain

Somewhat certain

Moderately certain

Very certain

Completely certain

DCE Intro

About your preferences for remote meetings...

Now we will ask you to choose between different ways that remote meetings for a research project could be organised. For the next questions:

Imagine you are being invited to take part in a new project as a public contributor/advisor. This will add to existing commitments you have, but you have time to take this on.

The team plans to hold regular meetings **remotely using video calls.** This means you will be asked to connect using a device (such as a phone/tablet/computer) to these meetings.

The video calls will be led by trained moderator who will make sure everyone is aware of the rules so that the meetings run smoothly.

Each question will show you two ways that the meetings could be organised. You will notice there are things that you like and dislike from each meeting type. We will ask you to choose the type of meeting that you would like best. You can also choose to not take part in the project.

Your answers will help the research team find out what is important to you when deciding to take part in a project.

Click **Next** to see how the questions will look like.

The questions will look like the one below. You choose a meeting by clicking on it. You can practice this now.

Click **Next** when you are ready to move on.

Meeting Type 1	Meeting Type 2	
You…<br>…attend **1 hr 30 min** long meetings with **no comfort break…**<br>…arranged during **working hours** and/or **evenings/weekends.**<br>…use your **own devices,** and receive **payment to cover internet and electricity costs** for the meetings.<br>…are given **instructions, online training and one-to-one support** on how to attend and participate.	You…<br>…attend **2 hr** long meetings with a **comfort break…**<br>…arranged during **working hours.**<br>…receive **all the devices you need,** and **payment to cover internet and electricity** costs for the meetings.<br>…are given **instructions** on how to attend and participate.	Don't take part in the project.
During these meetings…<br>…you must have your **camera on** and be ready to participate **at all times.**<br>…the moderator focuses on **ensuring they run smoothly** and making you **feel comfortable and confident** about contributing.	During these meetings…<br>…you can have your **camera off** and **step away when/if needed.**<br>…the moderator focuses on **ensuring they run smoothly.**	
After these meetings, you…<br>…receive **general** follow up that tells you how **the group's contributions** were taken on-board.	After these meetings, you…<br>…receive **personalised** follow up that tells you how **your contributions** were taken on-board.	

We will ask you **EIGHT** of these choice questions. As you move through the questions, at first glance the meeting types may appear the same, but you will notice they change slightly across questions.

Each question is separate from the others. Please choose what you would prefer to do in each question independently of what you answered previously.

Click **Next** to start the choice questions.

DCE Block 1

Which meeting type would you prefer?

Meeting Type 1	Meeting Type 2	
You… …attend **1 hr 30 min** long meetings with **no comfort break…** …arranged during **working hours** and/or **evenings/weekends.** …use your **own devices, internet and electricity** for the meetings. …are given **instructions, online training and one-to-one support** on how to attend and participate.	You… …attend **2 hr 30 min** long meetings with a **comfort break** and **a social activity…** …arranged during **working hours.** …use your **own devices,** and receive **payment to cover internet and electricity costs** for the meetings. …are given **instructions** on how to attend and participate.	Don't take part in the project.
During these meetings… …you can have your **camera off** and **step away when/if needed.** …the moderator focuses on **ensuring they run smoothly.**	During these meetings… …you must have your **camera on** and be ready to participate **at all times.** …the moderator focuses on **ensuring they run smoothly** and making you **feel comfortable and confident** about contributing.	
After these meetings, you… …receive **personalised** follow up that tells you how **your contributions** were taken on-board.	After these meetings, you… …receive **no follow up.**	

Which meeting type would you prefer?

Meeting Type 1	Meeting Type 2	
You…<br>…attend **2 hr 30 min** long meetings with a **comfort break** and **a social activity…**<br>…arranged during **working hours.**<br>…use your **own devices** and **internet,** and **receive payment to cover electricity costs** for the meetings.<br>…are given **instructions and online training** on how to attend and participate.	You…<br>…attend **1 hr 30 min** long meetings with **no comfort break…**<br>…arranged during **working hours** and/or **evenings/weekends.**<br>…use your **own devices, internet and electricity** for the meetings.<br>…are given **instructions** on how to attend and participate.	Don't take part in the project.
During these meetings…<br>…you can have your **camera off** and **step away when/if needed.**<br>…the moderator focuses on **ensuring they run smoothly.**	During these meetings…<br>…you must have your **camera on** and be ready to participate **at all times.**<br>…the moderator focuses on **ensuring they run smoothly** and making you **feel comfortable and confident** about contributing.	
After these meetings, you…<br>…receive **general** follow up that tells you how **the group's contributions** were taken on-board.	After these meetings, you…<br>…receive **personalised** follow up that tells you how **your contributions** were taken on-board.	

Which meeting type would you prefer?

Meeting Type 1	Meeting Type 2	
You…<br>…attend **1 hr 30 min** long meetings with **no comfort break…**<br>…arranged during **working hours** and/or **evenings/weekends.**<br>…use your **own devices,** and receive **payment to cover internet and electricity costs** for the meetings.<br>…are given **instructions and online training** on how to attend and participate.	You…<br>…attend **2 hr** long meetings with a **comfort break…**<br>…arranged during **working hours.**<br>…receive **all the devices you need,** and **payment to cover internet and electricity costs** for the meetings.<br>…are given **instructions** on how to attend and participate.	**Don't take part in the project.**
During these meetings…<br>…you can have your **camera off** and **step away when/if needed.**<br>…the moderator focuses on **ensuring they run smoothly** and making you **feel comfortable and confident** about **contributing.**	During these meetings…<br>…you must have your **camera on** and be ready to participate **at all times.**<br>…the moderator focuses on **ensuring they run smoothly.**	
After these meetings, you…<br>…receive **personalised** follow up that tells you how **your contributions** were taken **on-board.**	After these meetings, you…<br>…receive **general** follow up that tells you how **the group's contributions** were taken **on-board.**	

Which meeting type would you prefer?

Meeting Type 1	Meeting Type 2	
You…<br>…attend **1 hr 30 min** long meetings with **no comfort break…**<br>…arranged during **working hours.**<br>…use your **own devices** and **internet,** and **receive payment to cover electricity costs** for the meetings.<br>…are given **instructions, online training and one-to-one support** on how to attend and participate.	You…<br>…attend **2 hr 30 min** long meetings with a **comfort break** and a **social activity…**<br>…arranged during **working hours** and/or **evenings/weekends.**<br>…use your **own devices, internet and electricity** for the meetings.<br>…are given **instructions** on how to attend and participate.	Don't take part in the project.
During these meetings…<br>…you must have your **camera on** and be ready to participate **at all times.**<br>…the moderator focuses on **ensuring they run smoothly.**	During these meetings…<br>…you can have your **camera off** and **step away when/if needed.**<br>…the moderator focuses on **ensuring they run smoothly** and making you **feel comfortable and confident** about contributing.	
After these meetings, you…<br>…receive **general** follow up that tells you how **the group's contributions** were taken on-board.	After these meetings, you…<br>…receive **no follow up.**	

Which meeting type would you prefer?

Meeting Type 1	Meeting Type 2	
You… ...attend **2 hr** long meetings with a **comfort break…** ...arranged during **working hours** and/or **evenings/weekends.** ...use your **own devices, internet and electricity** for the meetings. ...are given **instructions and online training** on how to attend and participate.	You… ...attend **1 hr 30 min** long meetings with **no comfort break…** ...arranged during **working hours.** ...use your **own devices,** and receive **payment to cover internet and electricity costs** for the meetings. ...are given **instructions, online training and one-to-one support** on how to attend and participate.	**Don't take part in the project.**
During these meetings… ...you can have your **camera off** and **step away when/if needed.** ...the moderator focuses on **ensuring they run smoothly** and making you **feel comfortable and confident** about **contributing.**	During these meetings… ...you must have your **camera on** and be ready to participate **at all times.** ...the moderator focuses on **ensuring they run smoothly.**	
After these meetings, you… ...receive **general** follow up that tells you how **the group's contributions** were taken on-board.	After these meetings, you… ...receive **personalised** follow up that tells you how **your contributions** were taken on-board.	

Which meeting type would you prefer?

Meeting Type 1	Meeting Type 2	
You… …attend **2 hr** long meetings with a **comfort break…** …arranged during **working hours** and/or **evenings/weekends.** …receive **all the devices you need,** and **payment to cover internet and electricity costs** for the meetings. …are given **instructions and online training** on how to attend and participate.	You… …attend **2 hr 30 min** long meetings with a **comfort break** and **a social activity…** …arranged during **working hours.** …use your **own devices, internet and electricity** for the meetings. …are given **instructions, online training and one-to-one support** on how to attend and participate.	Don't take part in the project.
During these meetings… …you must have your **camera on** and be ready to participate **at all times.** …the moderator focuses on **ensuring they run smoothly** and making you **feel comfortable and confident** about contributing.	During these meetings… …you can have your **camera off** and **step away when/if needed.** …the moderator focuses on **ensuring they run smoothly.**	
After these meetings, you… …receive **personalised** follow up that tells you how **your contributions** were taken on-board.	After these meetings, you… …receive **general** follow up that tells you how **the group's contributions** were taken on-board.	

Which meeting type would you prefer?

Meeting Type 1

You…

…attend **2 hr 30 min** long meetings with a **comfort break** and **a social activity…**

…arranged during **working hours** and/or **evenings/weekends.**

…use your **own devices** and **internet,** and **receive payment to cover electricity costs** for the meetings.

…are given **instructions, online training and one-to-one support** on how to attend and participate.

During these meetings…

…you must have your **camera on** and be ready to participate **at all times.**

…the moderator focuses on **ensuring they run smoothly** and making you **feel comfortable and confident** about contributing.

After these meetings, you…

…receive **no follow up.**

Meeting Type 2

You…

…attend **1 hr 30 min** long meetings with **no comfort break…**

…arranged during **working hours.**

…receive **all the devices you need,** and **payment to cover internet and electricity costs** for the meetings.

…are given **instructions** on how to attend and participate.

During these meetings…

…you can have your **camera off** and **step away when/if needed.**

…the moderator focuses on **ensuring they run smoothly.**

After these meetings, you…

…receive **general** follow up that tells you how **the group's contributions** were taken on-board.

Don't take part in the project.

Which meeting type would you prefer?

Meeting Type 1	Meeting Type 2	
You…<br>…attend **2 hr 30 min** long meetings with a **comfort break** and **a social activity…**<br>…arranged during **working hours** and/or **evenings/weekends.**<br>…use your **own devices,** and receive **payment to cover internet and electricity costs** for the meetings.<br>…are given **instructions** on how to attend and participate.	You…<br>…attend **2 hr** long meetings with a **comfort break…**<br>…arranged during **working hours.**<br>…use your **own devices** and **internet,** and **receive payment to cover electricity** costs for the meetings.<br>…are given **instructions, online training and one-to-one support** on how to attend and participate.	Don't take part in the project.
During these meetings…<br>…you must have your **camera on** and be ready to participate **at all times.**<br>…the moderator focuses on **ensuring they run smoothly.**	During these meetings…<br>…you can have your **camera off** and **step away when/if needed.**<br>…the moderator focuses on **ensuring they run smoothly** and making you **feel comfortable and confident** about contributing.	
After these meetings, you…<br>…receive **general** follow up that tells you how **the group's contributions** were taken on-board.	After these meetings, you…<br>…receive **no follow up.**	

DCE Block 2

Which meeting type would you prefer?

Meeting Type 1	Meeting Type 2	
You…<br>…attend **2 hr** long meetings with a **comfort break…**<br>…arranged during **working hours.**<br>…use your **own devices,** and receive **payment to cover internet and electricity costs** for the meetings.<br>…are given **instructions** on how to attend and participate.	You…<br>…attend **2 hr 30 min** long meetings with a **comfort break** and **a social activity…**<br>…arranged during **working hours** and/or **evenings/weekends.**<br>…receive **all the devices you need,** and **payment to cover internet and electricity costs** for the meetings.<br>…are given **instructions and online training** on how to attend and participate.	**Don't take part in the project.**
During these meetings…<br>…you must have your **camera on** and be ready to participate **at all times.**<br>…the moderator focuses on **ensuring they run smoothly** and making you **feel comfortable and confident** about contributing.	During these meetings…<br>…you can have your **camera off** and **step away when/if needed.**<br>…the moderator focuses on **ensuring they run smoothly.**	
After these meetings, you…<br>…receive **general** follow up that tells you how **the group's contributions** were taken on-board.	After these meetings, you…<br>…receive **no follow up.**	

Which meeting type would you prefer?

Meeting Type 1	Meeting Type 2	
You…<br>…attend **2 hr** long meetings with a **comfort break…**<br>…arranged during **working hours** and/or **evenings/weekends.**<br>…receive **all the devices you need,** and **payment to cover internet and electricity costs** for the meetings.<br>…are given **instructions, online training and one-to-one support** on how to attend and participate.	You…<br>…attend **2 hr 30 min** long meetings with a **comfort break** and **a social activity…**<br>…arranged during **working hours.**<br>…use your **own devices** and **internet,** and **receive payment to cover electricity costs** for the meetings.<br>…are given **instructions and online training** on how to attend and participate.	Don't take part in the project.
During these meetings…<br>…you must have your **camera on** and be ready to participate **at all times.**<br>…the moderator focuses on **ensuring they run smoothly.**	During these meetings…<br>…you can have your **camera off** and **step away when/if needed.**<br>…the moderator focuses on **ensuring they run smoothly** and making you **feel comfortable and confident** about **contributing.**	
After these meetings, you…<br>…receive **no follow up.**	After these meetings, you…<br>…receive **personalised** follow up that tells you how **your contributions** were taken **on-board.**	

Which meeting type would you prefer?

Meeting Type 1	Meeting Type 2	
You…	You…	Don’t take part in the project.
…attend **1 hr 30 min** long meetings with **no comfort break…**	…attend **2 hr** long meetings with a **comfort break…**	
…arranged during **working hours.**	…arranged during **working hours** and/or **evenings/weekends.**	
…use your **own devices, internet and electricity** for the meetings.	…use your **own devices** and **internet,** and **receive payment to cover electricity** costs for the meetings.	
…are given **instructions and online training** on how to attend and participate.	…are given **instructions** on how to attend and participate.	
During these meetings…	During these meetings…	
…you must have your **camera on** and be ready to participate **at all times.**	…you can have your **camera off** and **step away when/if needed.**	
…the moderator focuses on **ensuring they run smoothly** and making you **feel comfortable and confident** about **contributing.**	…the moderator focuses on **ensuring they run smoothly.**	
After these meetings, you…	After these meetings, you…	
…receive **no follow up.**	…receive **personalised** follow up that tells you how **your contributions** were taken **on-board.**	

Which meeting type would you prefer?

Meeting Type 1	Meeting Type 2	
You…<br>…attend **1 hr 30 min** long meetings with **no comfort break…**<br>…arranged during **working hours** and/or **evenings/weekends.**<br>…use your **own devices** and **internet,** and **receive payment to cover electricity costs** for the meetings.<br>…are given **instructions** on how to attend and participate.	You…<br>…attend **2 hr** long meetings with a **comfort break…**<br>…arranged during **working hours.**<br>…use your **own devices,** and receive **payment to cover internet and electricity costs** for the meetings.<br>…are given **instructions and online training** on how to attend and participate.	**Don't take part in the project.**
During these meetings…<br>…you can have your **camera off** and **step away when/if needed.**<br>…the moderator focuses on **ensuring they run smoothly** and making you **feel comfortable and confident** about contributing.	During these meetings…<br>…you must have your **camera on** and be ready to participate **at all times.**<br>…the moderator focuses on **ensuring they run smoothly.**	
After these meetings, you…<br>…receive **general** follow up that tells you how **the group's contributions** were taken on-board.	After these meetings, you…<br>…receive **personalised** follow up that tells you how **your contributions** were taken on-board.	

Which meeting type would you prefer?

Meeting Type 1	Meeting Type 2	
You…<br>…attend **2 hr** long meetings with a **comfort break…**<br>…arranged during **working hours.**<br>…use your **own devices,** and receive **payment to cover internet and electricity costs** for the meetings.<br>…are given **instructions** on how to attend and participate.	You…<br>…attend **1 hr 30 min** long meetings with **no comfort break…**<br>…arranged during **working hours** and/or **evenings/weekends.**<br>…receive **all the devices you need,** and **payment to cover internet and electricity costs** for the meetings.<br>…are given **instructions and online training** on how to attend and participate.	**Don't take part in the project.**
During these meetings…<br>…you can have your **camera off** and **step away when/if needed.**<br>…the moderator focuses on **ensuring they run smoothly.**	During these meetings…<br>…you must have your **camera on** and be ready to participate **at all times.**<br>…the moderator focuses on **ensuring they run smoothly** and making you **feel comfortable and confident** about contributing.	
After these meetings, you…<br>…receive **no follow up.**	After these meetings, you…<br>…receive **general** follow up that tells you how **the group's contributions** were taken on-board.	

Which meeting type would you prefer?

Meeting Type 1	Meeting Type 2	
You…<br>…attend **2 hr 30 min** long meetings with a **comfort break** and **a social activity…**<br>…arranged during **working hours.**<br>…use your **own devices, internet and electricity** for the meetings.<br>…are given **instructions** on how to attend and participate.	You…<br>…attend **1 hr 30 min** long meetings with **no comfort break…**<br>…arranged during **working hours** and/or **evenings/weekends.**<br>…use your **own devices,** and receive **payment to cover internet and electricity costs** for the meetings.<br>…are given **instructions, online training and one-to-one support** on how to attend and participate.	Don't take part in the project.
During these meetings…<br>…you must have your **camera on** and be ready to participate **at all times.**<br>…the moderator focuses on **ensuring they run smoothly.**	During these meetings…<br>…you can have your **camera off** and **step away when/if needed.**<br>…the moderator focuses on **ensuring they run smoothly** and making you **feel comfortable and confident** about contributing.	
After these meetings, you…<br>…receive **personalised** follow up that tells you how **your contributions** were taken on-board.	After these meetings, you…<br>…receive **general** follow up that tells you how **the group's contributions** were taken on-board.	

Which meeting type would you prefer?

Meeting Type 1	Meeting Type 2	
You… ...attend **1 hr 30 min** long meetings **with no comfort break...** ...arranged during **working hours.** ...receive **all the devices you need,** and **payment to cover internet and electricity costs** for the meetings. ...are given **instructions** on how to attend and participate.	You… ...attend **2 hr** long meetings with a **comfort break...** ...arranged during **working hours** and/or **evenings/weekends.** ...use your **own devices, internet and electricity** for the meetings. ...are given **instructions, online training and one-to-one support** on how to attend and participate.	Don't take part in the project.
During these meetings… ...you can have your **camera off** and **step away when/if needed.** ...the moderator focuses on **ensuring they run smoothly.**	During these meetings… ...you must have your **camera on** and be ready to participate **at all times.** ...the moderator focuses on **ensuring they run smoothly** and making you **feel comfortable and confident** about contributing.	
After these meetings, you… ...receive **no follow up.**	After these meetings, you… ...receive **general** follow up that tells you how **the group's contributions** were taken on-board.	

Which meeting type would you prefer?

Meeting Type 1	Meeting Type 2	
You…<br>…attend **2 hr 30 min** long meetings with a **comfort break** and a **social activity…**<br>…arranged during **working hours.**<br>…receive **all the devices you need,** and **payment to cover internet and electricity costs** for the meetings.<br>…are given **instructions, online training and one-to-one support** on how to attend and participate.	You…<br>…attend **1 hr 30 min** long meetings with **no comfort break…**<br>…arranged during **working hours** and/or **evenings/weekends.**<br>…use your **own devices** and **internet,** and **receive payment to cover electricity costs** for the meetings.<br>…are given **instructions and online training** on how to attend and participate.	Don’t take part in the project.
During these meetings…<br>…you can have your **camera off** and **step away when/if needed.**<br>…the moderator focuses on **ensuring they run smoothly** and making you **feel comfortable and confident** about **contributing.**	During these meetings…<br>…you must have your **camera on** and be ready to participate **at all times.**<br>…the moderator focuses on **ensuring they run smoothly.**	
After these meetings, you…<br>…receive **personalised** follow up that tells you how **your contributions** were taken **on-board.**	After these meetings, you…<br>…receive **no follow up.**	

Socio

About you...

In these last few questions will ask about you and your household's characteristics. All of the following question have been included to help us to figure out how personal circumstances affect people's use of remote communication technologies.

How old are you in years?

What is your sex?

Female

Male

[This question is optional. You do not have to answer if you do not want to.] Is the gender you identify with the same as your sex registered at birth?

Yes

No, write in gender identity:

What is your ethnic group?

White

Mixed or multiple ethnic groups

Asian or Asian British

Black, Black British, Caribbean or African

Other ethnic group

Prefer not to say

What is your marital or civil partnership status?

Single

Married, in a registered civil partnership or co-habiting

Separated

Divorced

Widowed

Prefer not to say

Do you have caring any caring responsibilities for a child/children and/or another adult/s?

No

Yes

Prefer not to say

What is your highest level of education?

No educational qualifications

School - GCSE/O Level

School - A Level or equivalent

Professional qualification

Undergraduate degree

Postgraduate degree

Other qualification

Is English your first language?

Yes

No

What is your employment status/work situation/benefits status? *Please select all that apply.*

Working full-time

Working part-time

Working self employed (part/full time)

Unemployed

Not working because of ill health or disability

Retired

Education full-time/part-time

Looking after home/family

Full time carer

Other inactive

Prefer not to say

Including yourself, how many adults live in your household?

How many children (under 18) live in your household?

Which group represents your total household income including any benefits received and before any deductions? *Please select either weekly or annual income.*

Up to £99 weekly
£100 and up to £199 weekly
£200 and up to £299 weekly
£300 and up to £399 weekly
£400 and up to £499 weekly
£500 and up to £599 weekly
£600 and up to £699 weekly
£700 and up to £999 weekly
£1000 and above weekly
Prefer not to say
Up to £5,199 annual
£5,200 and up to £10,399 annual
£10,400 and up to £15,599 annual
£15,600 and up to £20,799 annual
£20,800 and up to £25,999 annual
£26,000 and up to £31,199 annual
£31,200 and up to £36,399 annual
£36,400 and up to £51,999 annual
£52,000 and above annual

Can you tell us which type of device you used to complete this survey?

Mobile phone	Tablet or iPad	Laptop computer	Desktop computer	Other (please give details)

Powered by Qualtrics
